# Proteomics-Based Approaches to Decipher the Molecular Strategies of *Botrytis cinerea*: A Review

**DOI:** 10.3390/jof11080584

**Published:** 2025-08-06

**Authors:** Olivier B. N. Coste, Almudena Escobar-Niño, Francisco Javier Fernández-Acero

**Affiliations:** Microbiology and Proteomics Laboratory, Institute for Viticulture and Agri-Food Research (IVAGRO), Faculty of Marine and Environmental Sciences, Department of Biomedicine, Biotechnology and Public Health, University of Cádiz, 11510 Puerto Real, Spain; olivier.coste@uca.es (O.B.N.C.); almudena.escobar@uca.es (A.E.-N.)

**Keywords:** *Botrytis cinerea*, proteomics, secretome, extracellular vesicles, plant-pathogen interaction, fungal virulence, pathogenesis, fungicide resistance, molecular mechanisms, plant defense

## Abstract

*Botrytis cinerea* is a highly versatile pathogenic fungus, causing significant damage across a wide range of plant species. A central focus of this review is the recent advances made through proteomics, an advanced molecular tool, in understanding the mechanisms of *B. cinerea* infection. Recent advances in mass spectrometry-based proteomics—including LC-MS/MS, iTRAQ, MALDI-TOF, and surface shaving—have enabled the in-depth characterization of *B. cinerea* subproteomes such as the secretome, surfactome, phosphoproteome, and extracellular vesicles, revealing condition-specific pathogenic mechanisms. Notably, in under a decade, the proportion of predicted proteins experimentally identified has increased from 10% to 52%, reflecting the rapid progress in proteomic capabilities. We explore how proteomic studies have significantly enhanced our knowledge of the fungus secretome and the role of extracellular vesicles (EVs), which play key roles in pathogenesis, by identifying secreted proteins—such as pH-responsive elements—that may serve as biomarkers and therapeutic targets. These technologies have also uncovered fine regulatory mechanisms across multiple levels of the fungal proteome, including post-translational modifications (PTMs), the phosphomembranome, and the surfactome, providing a more integrated view of its infection strategy. Moreover, proteomic approaches have contributed to a better understanding of host–pathogen interactions, including aspects of the plant’s defensive responses. Furthermore, this review discusses how proteomic data have helped to identify metabolic pathways affected by novel, more environmentally friendly antifungal compounds. A further update on the advances achieved in the field of proteomics discovery for the organism under consideration is provided in this paper, along with a perspective on emerging tools and future developments expected to accelerate research and improve targeted intervention strategies.

## 1. Introduction

### 1.1. Economic and Ecological Threat

*Botrytis cinerea* is a pathogenic fungus that affects a large spectrum of the plant kingdom. In the right environmental conditions, it can potentially cause damage to any plant tissue containing the right amount of moisture. Its success as a necrotrophic ascomycete fungus is due to its genetic plasticity and diversity, which offer a superior arsenal to infect hosts compared to fungi with a narrower host range [1]. This genetic diversity likely contributes to the fungus’s ability to adapt to diverse ecological niches [2].

One of the implications of this is that *B. cinerea* is among the most harmful postharvest pathogens for the fresh produce industry. In citrus, it accounts for 2–15% of total post-harvest rots after 2 months of storage and 30% of mandarin decays. In pome fruit, losses as high as 20–60% of total production due to gray mold are reported after an extended period of storage. Moreover, gray mold is the most important postharvest disease in grapes and can reach an incidence of up to 50% incidence within a month under cold storage [3].

*B. cinerea causes* significant postharvest losses by infecting fruits during storage and transport [4]. However, beyond its effects after harvest, it also affects living plants by inducing necrosis in vegetative and reproductive tissues, leading to flower abortion and reduced yield. In ornamental crops and fresh-cut flowers [5] —where both flowers and foliage represent key market components —managing Botrytis during the plant’s development is especially critical. Therefore, effective control strategies are needed throughout all stages of cultivation.

### 1.2. Unveiling Virulence Through Proteomics

Although several chemical compounds exist to control this fungus at plant, pre and postharvest levels, new resistant strains have been commonly described in the literature for many years [6,7,8]. It is thus of significant interest to deepen the comprehension of this organism to focus on sustainable disease-management strategies. The identification of novel therapeutic targets within diverse metabolic pathways has the potential to provide novel insights into the development of fungicide formulations. Proteomics is a valuable tool for the description of fungal metabolic pathways in different situations, since it enables the identification of a molecular phenotype at a given time. It is evident that these insights are of great value in two key areas. Firstly, they facilitate a more profound comprehension of the mechanisms through which existing chemicals, which have been proven to be effective against the fungus, act. Secondly, they serve to elucidate the fungal strategies employed to infect its host. This, in turn, paves the way for the identification of potential new molecular targets.

Extensive genomic studies of *Botrytis cinerea* have revealed valuable insights into its diverse tools for thriving in various ecological niches [9]. However, this genomic approach offers only a partial understanding of the system, as not all genes are actively expressed, and even when transcribed into mRNA, they may not necessarily be translated into functional proteins. Additional factors, such as protein stability over time, activation and deactivation cycles, and the reversible binding of side groups, influence the organism’s biological dynamics. Studying the proteome helps clarify which molecular components are functionally involved in a given phenotype. This approach is particularly valuable for investigating pathogens that exhibit phenotypic plasticity in response to varying environments or host conditions and develop sophisticated strategies to resist or evade chemical effects [10,11].

Therefore, proteomics approaches help us to know key proteins in the pathogenicity process, called virulence or pathogenicity factors. These factors should represent optimal targets for drug design, as their presence is essential for the development of the infection cycle [12]. By the inhibition of their activities, we should be able to decrease or control the disease, being the best approach for the development of new environmentally friendly botricides.

## 2. *B. cinerea* Proteomics Approaches

**Figure 1:**
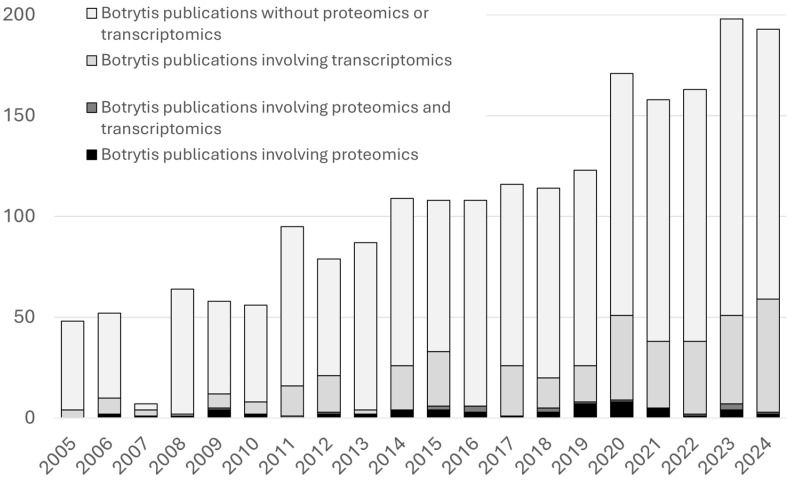
Number of Botrytis cinerea publications in PuMed in the last 20 years involving transcriptomics, proteomics, both, or none.

In the last extensive review about proteomics in *B. cinerea* [13] (2016), significant technical milestones had already been achieved, laying the foundation for a much faster elucidation of the fungal proteome. The publication of its genome in 2011 [2] and especially its subsequent quality improvement in 2017 [14] were crucial steps forward, enabling protein identification by comparing MS data directly with putative proteins encoded in the genome rather than relying on comparisons with other organisms. This approach is now widely used for large-scale *B. cinerea* protein identification in a single sample, known as ‘shotgun proteomics’, which allows for the detection of thousands of proteins and significantly accelerates research. However, despite the potential of proteomics to elucidate molecular mechanisms in *B. cinerea*, its application in the study of this pathogen remains limited. As illustrated in Figure 1 and Table 1, the number of studies employing proteomic approaches in *B. cinerea* is still relatively low compared to other methodologies. This review provides an update on the advances in *B. cinerea* proteomics that have been achieved since the publication of the previous review in 2016 [13]. Consequently, it will not address the subject in any depth but rather refer the reader to the aforementioned review for a comprehensive overview.

### 2.1. Subproteomes

To cope with the complexity and wide dynamic range of the total proteome, many proteomics studies focus on the enrichment of specific subsets of proteins—so-called subproteomes. This approach enhances the detection of low-abundance but functionally relevant proteins that might otherwise be masked by highly abundant species in whole-cell extracts [28]. By targeting subcellular compartments or functional classes of proteins (e.g., membrane, secreted, or nuclear proteins), researchers can improve analytical sensitivity and obtain a more focused understanding of specific biological processes, such as signaling, transport, or host–pathogen interactions. For example, surface or secreted subproteomes are particularly informative in studies of fungal virulence and host infection mechanisms, as they include the first proteins involved in host recognition and colonization.

#### 2.1.1. Secretome: A Key Virulence Compartment

The *B. cinerea* secretome has been the fungal subproteome with most contributions. Several proteomic descriptions have been developed to elucidate this crucial set of molecular tools in the fungal infection cycle. From the first description in 2009, these approaches used to work with proteomics of the first generation, normally based on 2DE gels and MALDI TOF/TOF, where most of the fungal proteins were masked by the huge amount of plant proteins.

Recently, a novel approach was developed to characterize the B. cinerea secretome during the early stages of plant infection in situ (0, 6, 12, and 24 h post-infection), comparing it to gene expression profiles from mycelium grown in vitro [17]. A total of 219 secreted proteins were identified by LC-MS, many of which were hydrolases. This study revealed that the expression pattern of these proteins differs significantly between in vitro and in vivo conditions. To assess their functional relevance, nine genes encoding putative virulence factors were deleted. While the mutants showed no major growth defects in vitro, several displayed reduced virulence during apple infection. These results underscore the importance of investigating the secretome under in vivo conditions, as it provides critical insight into the infection strategy and virulence mechanisms of the fungus.

The modulation of environmental pH represents a critical determinant in the deployment of virulence factors by *Botrytis cinerea*, particularly through its influence on the composition and activity of the fungal secretome. Recent studies have elucidated the role of specific genetic regulators—namely the VELVET complex and the BcPacC transcription factor—in mediating pH-dependent pathogenicity mechanisms, with quantitative proteomics providing detailed insight into secreted protein dynamics under variable pH conditions.

Müller et al. explored the impact of VELVET complex inactivation on *B. cinerea* pathogenicity and secretory behavior using a refined N^15^ metabolic labeling strategy [21]. In this approach, tomato leaves were enriched with N^15^ and infected with *B. cinerea*, and a defined quantity of N^14^-labeled standard secretome was spiked into each sample. Proteins were digested, and quantification was based on the integration of N^14^/N^15^ peak pairs and their normalized area ratios. This technique allowed the sensitive and accurate quantification of 174 fungal and 141 plant proteins from infected tissues, revealing a significant reduction in secreted fungal proteins in VELVET-deficient mutants. Notably, functional categories such as carbohydrate-active enzymes (CAZymes) and proteases were markedly downregulated in mutants, correlating with impaired acidification of the host environment and reduced virulence. Restoration of low pH through artificial acidification rescued CAZyme expression and partially restored infectivity, but protease expression remained suppressed, suggesting direct regulation by the VELVET complex beyond pH-mediated control.

Similarly, the role of BcPacC, a key transcription factor in fungal pH-sensing pathways, has been characterized through comparative secretomic analysis of wild-type and BcPacC-deletion mutants grown at two pH conditions (pH 5 and pH 7) [18]. In the absence of PacC, *B. cinerea* exhibits a significant defect in radial growth on complex media at a pH equal to or greater than 6.0. Proteomic profiling revealed that at neutral pH, the mutant exhibited a significantly reduced secretion of cell-wall-degrading enzymes (CWDEs) compared to the wild type, impairing the infection process. However, under acidic conditions, the deletion had no measurable effect on pathogenicity. This suggests that BcPacC is not a direct virulence regulator but may facilitate acidification through the control of oxalic acid or reactive oxygen species (ROS) production, which are necessary to establish a favorable low-pH environment for virulence factor deployment. Additionally, this study identified BcPacC-independent enzymes the expression of which was pH-regulated. For instance, several plant cell wall polysaccharide-degrading enzymes were upregulated at pH 7, whereas tripeptidyl peptidases and glyoxal oxidases were more abundant at pH 5, indicating the existence of alternative pH-responsive regulatory circuits.

Taken together, these studies underscore a complex interplay between pH modulation and secretome composition in *B. cinerea*. While pH serves as an environmental cue shaping the expression of virulence-associated proteins, genetic regulators such as the VELVET complex and BcPacC mediate the fungal response to this cue through both direct transcriptional control and indirect modulation of the extracellular environment. The study of mutant strains through transcriptomics and proteomics has been instrumental in dissecting these mechanisms, offering a high-resolution view of the fungal secretion landscape under varying pH conditions. These insights are critical for understanding fungal pathogenesis and may inform future strategies for disease control based on the disruption of pH-regulated virulence programs.

The study of the BcactA mutant—an actin-deficient mutant—highlighted the essential role of the actin cytoskeleton in the fungal secretory pathway [25]. The actin-deficient strain exhibited significantly reduced virulence, and an iTRAQ-based proteomic analysis of its secretome revealed a marked decrease in the abundance of cell-wall-degrading enzymes (CWDEs) among other factors. Among the 11 differentially regulated CWDEs, two—cellobiohydrolase (BcCBH) and β-endoglucanase (BcEG)—were identified as key virulence factors.

The authors suggest that this phenotype can be explained by the disruption of actin-mediated vesicle trafficking. In filamentous fungi, actin cables extend from the plasma membrane into the cell and serve as tracks for the targeted delivery of secretory vesicles. In pathogenic species, these vesicles transport virulence-associated proteins that are essential for host tissue penetration and colonization. Therefore, deletion of actA likely impairs the efficient delivery of these effectors to the extracellular space, resulting in a weakened infection process.

#### 2.1.2. Extracellular Vesicles: Emerging Players in Fungal Pathogenicity

The secretome, which comprises all proteins released by the fungus during infection, plays a key role in modulating plant responses and facilitating pathogenesis. The composition and functional relevance of the *B. cinerea* secretome has been previously described by different proteomics approaches [13]. However, a key component of this process was poorly described: the relevance of extracellular vesicles in the biology of *B. cinerea*. Extracellular vesicles (EVs) are nano-sized, membranous structures secreted into the extracellular space, exhibiting diverse sizes, contents, and surface markers, released from cells under normal and pathological conditions [29].

In a first study, *B. cinerea* EVs were obtained using a synthetic solid medium as a culture condition and isolation by ultracentrifugation on a density gradient medium [11]. For detailed information on the protocols used to minimize contamination from culture medium or cellular debris during EV isolation and proteome analysis, readers are referred to the original study cited herein. A total of 2461 proteins were identified; detected EVs proteins fall into two main categories: cytosolic or membrane-anchored. Although well-established EV markers were identified, only one virulence-associated protein and one cell-death-inducing protein were detected. While no canonical effector proteins were identified, five proteins exhibited predicted structural similarities to known fungal effectors. A wide range of enzymes like lyases, hydrolases, and proteases were not considered in this work as virulence factors.

In a second publication, a wider approach to *B. cinerea* extracellular vesicles was realized by comparing different virulence stages, using glucose (GLU) as a sole carbon source as a constitutive stage and deproteinized tomato cell walls (TCW) to induce fungal virulence [16] to identify differences in the EV proteome. Extracellular vesicles were isolated from the supernatant of a 5-day-old *B. cinerea* culture by ultracentrifugation. The presence of three vesicle morphotypes ranging from 30 to 130 nm was confirmed by Transmission Electron Microscopy, and they were generally larger than the EVs isolated from the GLU supplemented medium. A rigorous protein extraction was subsequently performed on the EVs, and trypsin was digested for further LC-MS/MS analysis.

In brief, five proteins were identified as exclusive EVs in both conditions (GLU and TCW), and they were presented as good markers for EVs of *B. cinerea* in any other assayed condition. The gene ontology analysis showed an enrichment of the proteins related to cell wall metabolism and proteinases excreted by the TCW conditions both in EVs and in the supernatant. This demonstrates that both conventional (secreted proteins) and unconventional (EVs) secretion pathways cooperate during infection pathways of *Botrytis cinerea*. The KEGG analysis also demonstrated the presence of multiple virulence factors in the EVs, such as pectin degradation, nucleotide sugar biosynthesis, redox state, biotin production, cofactor metabolism, and signaling, corroborating that EVs are part of the strategy of *Botrytis cinerea* to infect the host.

These findings highlight condition-specific EV markers, with distinct protein profiles under constitutive (GLU) and virulence-induced (TCW) conditions, suggesting a dynamic adaptation of vesicle content during infection.

The discrepancies observed between the two studies regarding the virulence-related content of *B. cinerea* extracellular vesicles (EVs) can be attributed to both the culture conditions and the methodological approaches used to analyze the EV proteomes. In the first study, EVs were obtained from fungi grown on a synthetic solid medium, likely providing a nutrient-rich, non-inductive environment that does not strongly activate the pathogen’s virulence program. As a result, the EVs predominantly contained cytosolic and membrane proteins, with only minimal detection of known virulence factors and no canonical effectors according to EffectorP predictions. In contrast, the second study applied a more biologically relevant comparison using glucose (constitutive stage) and deproteinized tomato cell wall (TCW, a virulence-inducing condition), which mimics the host environment and triggers infection-related pathways. This led to the identification of numerous virulence-associated proteins in EVs, including enzymes involved in cell wall degradation, redox control, and signaling, as revealed by KEGG and GO analyses. Furthermore, the methodologies used for functional prediction differed notably: while the first study relied on structural and effector-specific prediction tools (e.g., EffectorP, ApoplastP), the second focused on pathway enrichment and broader functional annotation, allowing for the identification of virulence factors not classically classified as effectors. These differences not only reflect the methodological bias inherent to each approach, but also highlight the dynamic and condition-specific nature of EV cargo. Importantly, they open new avenues to understand how *B. cinerea* regulates the selective inclusion of virulence factors into EVs in response to host-derived signals, and how this contributes to successful infection.

#### 2.1.3. Surface-Exposed Proteins: The Frontline of Molecular Interaction

Surface proteins (i.e., membrane proteins with external domains) are the first line of interaction with the plant, so their understanding is particularly relevant to designing new control agents. The so-called “shaving” method is a proteomic technique that allows the trypsinization of the one organism’s proteins that are exposed to the medium, leaving the rest of the proteins untouched. The generated peptides are then analyzed by HPLC-MS/MS. In an experiment led by Escobar-Niño et al. (2021) [22], the surfactome of *B. cinerea* was described for the first time. Several carbon sources and times of infection were assayed, finding singular expression patterns.

The surfactome of *Botrytis cinerea* emerges as a critical interface in host–pathogen interactions, functioning not only as a structural barrier but also as a sensory and regulatory platform for the activation of virulence. This surface-associated subproteome includes a wide array of proteins involved in nutrient uptake, signal transduction, enzymatic activity, and environmental sensing. Notably, several proteins identified under virulence-inducing conditions (TCW) stand out as potential molecular targets for antifungal intervention. Among them, the small heat shock protein (A0A384J6V0) and the multiprotein-bridging factor 1 (MBF1; A0A384JRV2) are particularly relevant due to their known roles in stress response and transcriptional regulation in other pathogenic fungi. The spliceosomal component CDC40 (A0A384J4A8) also appears under early virulence-inducing conditions and may play a role in small RNA-mediated host–pathogen communication, a mechanism increasingly recognized in cross-kingdom RNA interference [30,31]. Additionally, enzymes such as delta-aminolevulinic acid dehydratase and a PKS-domain-containing protein were identified on the fungal surface, potentially linking extracellular localization to secondary metabolite biosynthesis and toxin regulation. These findings underscore the functional complexity of the surfactome and open new avenues for research aimed at disrupting early virulence signaling or targeting specific components of the surface proteome to control *B. cinerea* infections.

#### 2.1.4. Post-Translational Modifications (PTMs): Fine-Tuning Fungal Proteins

In proteomics, an approach that is still rarely adopted in the phytopathogen studies is the description of post-translational modifications. Among them, phosphorylation is of particular importance due to the role it plays in signal transduction for membrane proteins and hence in the virulence. If these studies are only few in number, the overlapping analysis of several subproteomes is even rarer. The analysis of phosphorylated membrane proteins was described by LC-MS/MS [24]. From this approach, 1112 phosphoproteins out of the membranome of the fungus grown under two different carbohydrate sources were detected. Interestingly, the phosphorylation pattern of seven proteins was found to be different amongst the two carbon source conditions, presenting different phosphopeptides and/or different phosphorylation sites. The authors underlined that two out of these seven proteins were involved in pyruvate metabolism. This suggests that post-translational modifications (PTMs) on their own can modulate the infection process in response to a specific host and that pyruvate metabolism plays a role in the host adaptation of the pathogen.

In a subsequent study of this group, a wider analysis of the fungus proteome showed that the cluster diversity of the phosphomembranome was upregulated under growth in tomato cell wall supplement medium versus glucose medium [27]. Interestingly, the diversity of the rest of the proteome (i.e., secretome, membranome, and phosphoproteome) was higher in the glucose-supplemented medium. This confirms that the phosphomembranome and the alterations of phosphorylation are key in the adaptation of the fungus to the host.

Although distinct in origin, both the surfactome and the phosphomembranome reveal complementary aspects of membrane-associated virulence mechanisms, with phosphorylation adding an additional regulatory layer to surface-exposed proteins. Under virulence-inducing conditions (TCW), *Botrytis cinerea* exhibits distinct phosphorylation patterns in key membrane-associated proteins, notably affecting pathways involved in unfolded protein response (UPR), ER-associated degradation (ERAD), oxidative stress, and programmed cell death. Specific phosphorylation events under TCW activate signaling components such as MAPKs (e.g., Sak1, Bos5), small GTPases (e.g., Cla4), and calcium-mediated cascades (e.g., BcCRZ1), promoting a cellular state primed for host invasion and environmental adaptation. These post-translational modifications reveal a tightly regulated mechanism by which the fungus orchestrates its pathogenic strategy. The identification of these condition-dependent phosphoproteins provides valuable targets within central metabolic and stress-related pathways, opening new avenues for the development of selective fungicides aimed at disrupting the infection process.

### 2.2. Proteomic Insights into B. cinerea–Host Interactions

This section reviews proteomic studies of the host plant’s response to *B. cinerea* infection. Understanding and studying the host’s response is key to revealing defense mechanisms and opens the door to analyzing mixed proteomes from both host and pathogen, enabling a more comprehensive understanding of their molecular interaction.

Liu et al. [30] investigated the infection process of *B. cinerea* in kiwifruit using LC-ESI-MS/MS with iTRAQ-labeled samples, identifying 196 differentially accumulated proteins (DAPs) between infected and control fruits. Most DAPs were associated with metabolic pathways, plant–pathogen interactions, and biosynthesis. Notably, myosin proteins involved in early defense responses were confirmed to play a protective role, while polygalacturonase-inhibitor proteins (PGIPs), which inhibit cell-wall-degrading enzymes, were also upregulated, contributing to resistance. Alterations in the MAPK signaling pathway were observed, with some components upregulated and others downregulated, suggesting that *B. cinerea* may interfere with host defense cascades. Additionally, differential regulation of ubiquitin–proteasome system proteins indicates possible pathogen manipulation of programmed cell death mechanisms. Pathogenesis-related proteins with leucine-rich repeat and latex-like domains were increased, whereas thaumatin-like proteins were downregulated. Heat shock proteins linked to jasmonic acid-mediated defense were also upregulated. Changes in reactive oxygen species (ROS) pathway proteins suggest a complex modulation of oxidative stress responses by the host, potentially influenced by the pathogen. The use of iTRAQ enabled a detailed and quantitative analysis, providing new insights into the molecular pathways activated and modulated during *B. cinerea* infection [19].

Another interesting approach to improve our understanding of the specific defense system triggered by a fungal infection is to use a Pathogen-Associated Molecular Pattern (PAMP) compound isolated from *B. cinerea* and observe the molecular response of the host. In a complementary approach, a group of researchers used a cerato-platanin, a secreted non-catalytic fungal enzyme and virulence factor obtained by heterologous expression, to study the molecular response of *Arabidopsis thaliana*. The difference in protein translation was assessed by 2D-gel electroforesis and MALDI-TOF analysis. A total of 94 proteins were found to be differentially expressed between the treatment and the control. It was shown that under incubation with the PAMP, the plant downregulated photosynthesis and activated its stress response system. As expected, reactive oxygen species (ROS) were activated as a defense response, as well as the corresponding self-protective ROS-scavenging proteins like catalases. The glutathione (GSH) is clearly activated with seven proteins upregulated and three downregulated. Activation of this pathway is related to the homeostasis maintained in oxidative conditions.

In this work, it is also interesting to find that even without the presence of the fungus, the plant activates proteins, among which are lectin-like proteins, usually related with fungus elements like chitin or oligogalacturonides.

This approach of using a single *B. cinerea* protein to dissect the host response is particularly valuable, as it allows precise characterization of the specific role of this protein in the immune reaction. Conversely, a reciprocal strategy could be envisaged, where the modulation of *B. cinerea* is studied in response to molecules derived from the host immune system, providing deeper insights into the dynamic molecular dialogue between pathogen and host.

Directly studying the interaction between *B. cinerea* and its host at the proteomic level provides deep and dynamic insights into the defense and counter-defense mechanisms deployed during infection. This approach offers valuable information on the temporal and spatial regulation of key proteins in both the pathogen and the plant, facilitating the identification of molecular targets for the development of more specific and effective control strategies. Moreover, such studies are essential to validate and refine in vitro or in silico models that aim to mimic the host–pathogen interaction. However, this technique faces significant challenges, including the biological complexity of the system, the overlap and similarity of proteins between both species, and the difficulty in distinguishing fungal proteins from host proteins in mixed samples. Additionally, biological variability, the physiological state of the host, and heterogeneity in responses across different tissues or cell types complicate data interpretation. Finally, the sensitivity and resolution required to detect subtle changes in low-abundance or post-translationally modified proteins remain technical hurdles. Despite these limitations, advances in separation, labeling, and high-resolution mass spectrometry techniques, such as iTRAQ, continue to improve our ability to unravel the complex host–pathogen interactions in important systems like *B. cinerea* and its plant hosts.

### 2.3. Functional Profiling of Botryticides Through Advanced Proteomics

The unisite botryticides can be classified into five main categories, which classify them by their molecular targets in the fungus cell. The first group of chemicals used for fungicide development are the cytoskeleton inhibitors. These are compounds that generally bind the cytoskeleton of the fungus, impairing proper cell division. The second group affects the osmoregulation of the plant, including the high-osmolarity glycerol (HOG) pathway, which eventually inhibits the conidial germination and the mycelial growth [32]. The third group includes inhibitors of amino acid biosynthesis, especially methionine. The fourth group inhibits the biosynthesis of ergosterol, which is a compound specific to the fungal kingdom. The fifth group is the one affecting cellular respiration and energy production. In this group, three botryticide types have been registered: complex II inhibitors (SDHIs), complex III (QoIs), and uncouplers of oxidative phosphorylation [6].

Wuyiencin is an antibiotic produced by *Streptomyces albulus* var. wuyiensis, which has a botryticide effect. A study of the effect of this molecule was performed by iTRAQ labeling LC-MS/MS. Over the 3816 proteins identified, 155 were found to be upregulated and 166 were found to be downregulated. To confirm the altered pathways, a subsequent Parallel Reaction Monitoring (PRM) analysis was performed on 50 different proteins. This approach validated the upregulation of 14 proteins—mainly those related to carbohydrate metabolism and cell wall stabilization—and the downregulation of 13 proteins involved in energy metabolism, nucleotide and protein synthesis, and the biosynthesis of stress- and decay-related compounds in plants. The remaining proteins did not yield consistent results.

In brief, wuyiencin was shown to upregulate carbohydrate metabolism associated proteins and downregulate proteins, amino acids, and nucleotide biosynthesis. This could indicate nutritive restrictions similarly to a starvation scenario. Overall, it is inferred that the differentially regulated proteins influence several key processes: the stress response was affected by the downregulation of oxidoreductases; fungal virulence was impacted through modulation of the abscisic acid pathway and downregulation of endopolygalacturonase; and mycelial growth, cell wall structure, and spore germination were associated with the upregulation of aspartic protease and esterase enzymes. This study lays the groundwork for understanding the molecular mechanisms underlying the effect of this antibiotic and highlights potential novel target pathways for disease control; however, further research is required to elucidate the precise molecular mechanisms involved [23].

The effect of an antimicrobial peptide belonging to the CSαβ-defensin family (cysteine-stabilized α-helix β-sheet) and derived from a mutagenesis-engineered analogue of heliomycin was investigated. The fungal proteome was analyzed through a dual approach: (i) molecular fingerprinting of growth inhibition using MALDI-MS, and (ii) bottom-up proteomic profiling via Nano-LC-ESI-MS/MS. The MALDI-MS approach enabled a rapid assessment of the peptide’s inhibitory effect, revealing a dose-dependent modulation of spectral peaks, particularly in the m/z range of 4000–6000. These molecular changes were correlated with experimentally observed growth inhibition. This type of assay may serve as a useful tool for the rapid detection of molecular alterations induced by antimicrobial agents.

In parallel, in-depth proteomic analysis was conducted to elucidate the molecular mechanisms underlying the peptide’s action. Based on previously defined selection criteria, six cellular pathways were found to be significantly affected by the treatment: spliceosome, ribosome, protein processing in the endoplasmic reticulum, endocytosis, MAPK signaling pathway, and oxidative phosphorylation. Notably, the disruption of cell wall integrity pathways has been reported for other defensins, supporting the observations in this study. The authors propose that the antimicrobial activity may involve interactions with lipid components of the fungal membrane, particularly glucosylceramides [33].

In summary, while wuyiencin and defensin antifungal agents share some common targets with traditional chemical fungicides—particularly pathways related to cell membrane integrity and energy metabolism—they also exert broader and distinct effects, including modulation of RNA processing and global metabolic adjustments, highlighting their potential as complementary tools in fungal disease control.

### 2.4. Synthesis of Proteomic Advances

The infection strategy of *Botrytis cinerea* is a finely regulated, multifaceted process that integrates environmental sensing, metabolic reprogramming, secretion of virulence factors, and active host manipulation. Proteomic analyses have been essential to unravel how this dynamic adaptation unfolds through distinct subproteomes and post-translational modifications. Upon contact with host tissues—particularly polysaccharide-rich structures like the plant cell wall—*B. cinerea* perceives physical and chemical cues that trigger its virulence program. This leads to rapid reconfiguration of its membrane phosphoproteome, with condition-specific phosphorylation of signaling proteins such as MAPKs (e.g., Sak1), small GTPases (e.g., Cla4), and calcium-dependent regulators (e.g., BcCRZ1).

Downstream of this signaling, the pathogen undergoes profound metabolic reprogramming, including phosphorylation-dependent modulation of central enzymes like pyruvate dehydrogenase and acetyl-CoA carboxylase. These changes redirect carbon fluxes toward the biosynthesis of lipids, toxins, and infection-related structures. In parallel, *B. cinerea* secretes a broad arsenal of virulence factors via both conventional (secretome) and unconventional (extracellular vesicles, EVs) pathways. These include cell-wall-degrading enzymes, oxidoreductases, and signaling proteins that weaken plant defenses and reconfigure the host redox environment. EVs, in particular, exhibit condition-specific cargo profiles and may serve to deliver non-canonical effectors during infection.

At the interface with the host, surface-associated proteins (the surfactome) mediate nutrient uptake, signal transduction, and environmental sensing, potentially contributing to cross-kingdom RNA interference. Moreover, *B. cinerea* actively modifies the apoplast, acidifying the local environment through organic acid secretion—a process dependent on regulators such as BcPacC. As infection progresses, the composition of all subproteomes continues to evolve, reflecting activation of stress responses, autophagy, and programmed cell death in both host and pathogen. The fungus counters plant defenses—including jasmonate signaling and CWDE inhibitors—through fine-tuned secretion and manipulation of host pathways, highlighting a dynamic, reciprocal battle at the molecular level.

These recent proteomic studies represent a significant advance over previous knowledge, which focused primarily on a limited number of secreted enzymes and classical virulence traits identified in vitro [24,34,35,36,37,38]. By enabling the targeted analysis of subproteomes such as the secretome, phosphomembranome, and EVs under host-mimicking conditions, these approaches have revealed the condition-dependent nature of virulence regulation and uncovered key signaling and metabolic pathways involved in host adaptation. Moreover, the identification of post-translational modifications and the discovery of an EV-mediated protein export offer novel insights into how *B. cinerea* fine-tunes its infection machinery. Collectively, these findings provide a more detailed and ecologically relevant view of fungal pathogenesis and open new avenues for the development of highly specific antifungal strategies.

## 3. Discovered Proteome Overview

### 3.1. B. cinerea Proteome Coverage

Not too long ago, the *B. cinerea* protein identification was largely based on genome similarity searches with other fungi. The development of *B. cinerea* sequencing projects has helped to improve protein identification procedures [39]. The coverage of the proteome relative to the genome of *B. cinerea* has steadily increased over the years, rising from 4.4–6.6% in 2016 [13] to 54% in 2021, according to the latest available data [27]. This indicates that more than half of the predicted proteins have been identified through proteomics experiments and that the speed of protein discoveries has dramatically increased since the last extensive revision in 2016. This has allowed us to identify unequivocally molecular mechanisms in the fungus implicated in fungicide response or host infection, which is already contributing to the control of this pathogen.

For this calculation, the latest genome assembly of *B. cinerea* strain B05.10 was used. In our study, we incorporated the most recent protein lists and identified 6794 out of the 13,037 predicted proteins, achieving a coverage of 52.1%. This is 2% lower than previously estimated, which can be explained by the fact that the earlier estimate was based on a comparison of detected proteins against the number of coding genome sequences, 11,707 [14], whereas our current comparison is made against the total number of predicted proteins following the UniProtKB Database.

Initially, we aimed to estimate protein coverage by building upon a previous study [27], incorporating newly identified proteins reported in recent publications. The earlier estimate was based on NCBI GI numbers for *B. cinerea* strain B05.10. However, unifying protein lists into a single identifier format proved challenging due to the distinct data sources used in this study. The latest protein identifications were assigned either UniProtKB or FungiEnsembl codes. Although we aimed to convert all identifiers to the UniProtKB format, this process sometimes led to mismatches, assigning different ID codes to the same protein due to variations in the reference strains used for identification (e.g., *B. cinerea* vs. *B. fuckeliana*). Using this approach, an equivocal identification rate of 72% turned out to be an artificial overestimate. To address this issue, we performed a BLAST search of the entire set of 8531 sequences against the predicted proteins of *B. cinerea* B05.10. After removing duplicates, we identified 6794 unique proteins. This refined dataset was subsequently used for gene ontology analysis.

Although a lower percentage was ultimately found compared to 2021, we consider this result more robust, as it is based on a comparison with the proteome of a single strain (*B05.10*) and accounts for the number of predicted proteins rather than just predicted genes. Notably, some proteins identified before 2021 may not be included in this count due to the lack of ID correspondence. These findings emphasize the need to improve alignment between protein databases.

### 3.2. Gene Ontology Analysis

**Figure 2:**
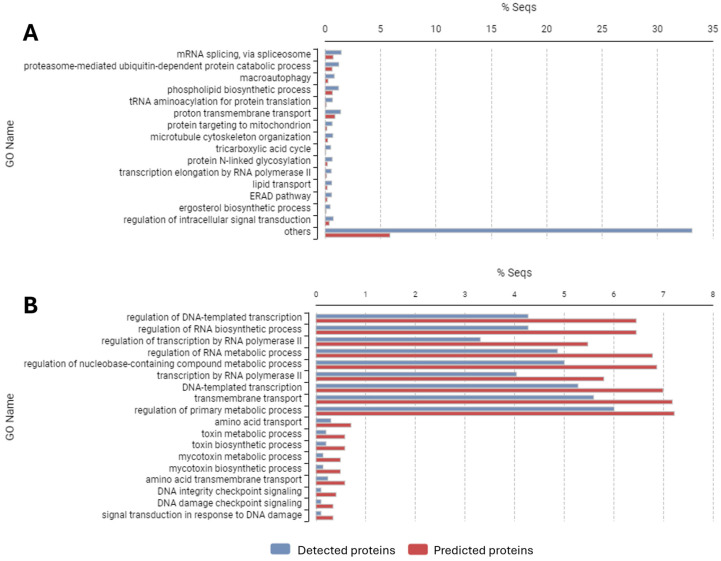
Biological process gene ontology enrichment bar chart of the whole identified *B. cinerea* proteins. (**A**) Overrepresented categories. (**B**) Underrepresented categories. Detected proteins: complete set of identified proteins among all proteomic approaches; predicted proteins: whole genome complete set of predicted proteins. Enrichment analysis was performed using the functional analysis section of the software OmicsBox 3.0.29.

To better understand which parts of the *B. cinerea* proteome remain unexplored, we performed a gene ontology (GO) analysis using the complete set of proteins identified so far through mass spectrometry across multiple published studies and compared them to the predicted proteome using the enrichment functionality of the OmicsBox 3.0.29 software (BioBam Bioinformatics). This comprehensive comparison revealed significant differences in the representation of functional categories (Figure 2).

Overrepresented categories in the detected proteome (Figure 2A) include RNA splicing, macroautophagy, phospholipid biosynthesis, and proton transmembrane transport, with a notable proportion classified as “other.” In contrast, 18 biological process categories are underrepresented (Figure 2B), including key functions such as transcription, transmembrane transport, primary metabolism, amino acid biosynthesis, DNA damage response, and mycotoxin production.

The overrepresentation of RNA splicing factors highlights the potential importance of post-transcriptional regulation in *B. cinerea*. The enrichment of proteins involved in macroautophagy suggests that many of the proteomic datasets analyzed may have captured stress conditions where cellular recycling mechanisms are activated [40]. The prominence of phospholipid biosynthetic pathways could indicate active membrane remodeling, possibly in response to environmental changes or different developmental stages of the fungus. Additionally, the overrepresentation of proton transmembrane transport proteins suggests a significant role in cellular energetics and pH regulation under the studied conditions. Notably, the category “other” is substantially overrepresented because it encompasses a vast number of processes beyond the 15 highly divergent biological processes discussed, with a total of 692 overrepresented categories.

The underrepresentation of transcription-related proteins may stem from their low abundance, as transcription factors and nuclear regulatory proteins are often difficult to detect using standard proteomic techniques. Similarly, transmembrane transporters are typically hydrophobic, posing challenges for solubilization and subsequent identification. The reduced presence of proteins involved in primary metabolism and amino acid metabolism suggests that the proteomic studies analyzed have predominantly captured conditions where *B. cinerea* is undergoing physiological transitions rather than a basal metabolic state. Additionally, the low representation of proteins related to mycotoxin biosynthesis may indicate that the experimental conditions used in the reviewed studies were not optimized for mycotoxin production, as these pathways are often activated under specific stress conditions or during host interactions. The underrepresentation of DNA damage checkpoint signaling proteins suggests that DNA repair mechanisms may be less active or challenging to detect in the analyzed proteomic datasets.

These findings suggest potential biases in proteomic methodologies, as hydrophobic and low-abundance proteins tend to be underrepresented in standard mass spectrometry-based analyses. Moreover, the experimental conditions of the reviewed studies may have primarily focused on stress responses and pathogenicity rather than the basal metabolic state of *B. cinerea*.

In addition, uncertainties remain regarding the accuracy and completeness of the predicted genome sequence, which may affect the identification and annotation of proteins. The potential expression of a cryptic genome—genes activated only under specific or rare conditions—also complicates the detection of the full proteome. Furthermore, overlapping or isoform-specific proteins pose additional challenges for unambiguous identification.

The observed differences between detected and predicted proteins also emphasize the potential relevance of post-transcriptional regulation and membrane dynamics in fungal adaptation.

Future studies aiming for a more comprehensive representation of *B. cinerea*’s functional proteome should consider optimizing experimental conditions to capture a broader spectrum of biological states, including those conducive to primary metabolism and mycotoxin biosynthesis. Furthermore, alternative proteomic strategies, such as targeted enrichment of membrane proteins and nuclear factors, could help mitigate technical biases and improve the detection of underrepresented functional categories.

## 4. Expectations for Future Developments

The proteome is a dynamic entity that changes with developmental stage and environmental conditions. As a complementary approach to genomics, transcriptomics, metabolomics, and other omics technologies, proteomics contributes to elucidating the functional mechanisms underlying the behavior of this organism. The proteome is not only dynamic in terms of presence/absence or abundance but also in the regulation of protein activity through post-translational modifications (PTMs)—a topic that has been scarcely explored so far. Further investigation into PTMs could significantly enhance our understanding of molecular mechanisms and protein–protein interactions.

An important yet still underexplored aspect in *B. cinerea* proteomics is the comparison between in vitro and in planta fungal proteomes. Fungal pathogens frequently undergo significant proteomic changes during host infection compared to axenic culture, reflecting specific adaptations to host environments and defense mechanisms. These dynamic changes are key to understanding fungal pathogenicity and developing targeted antifungal strategies. However, for *B. cinerea*, comprehensive in planta proteomic studies remain limited, highlighting a major gap and opportunity for future research.

In parallel, several promising proteomic technologies remain underexplored in *B. cinerea* research. Label-free quantitative proteomics, for example, offers a cost-effective and scalable approach to detect subtle proteome modulations across conditions or strains. Similarly, techniques such as thermal proteome profiling (TPP) [41] or limited proteolysis-coupled mass spectrometry (LiP-MS) [42,43] could help uncover protein structural shifts and interactions under stress or infection conditions. Advances in the enrichment and isolation of subproteomes—such as surface-exposed proteins and extracellular vesicles—through refined biotinylation protocols or nanoparticle-based capture systems will further improve specificity and coverage [43]. Incorporating these approaches into future studies would address current blind spots in *B. cinerea* proteomics and support a more comprehensive mapping of its molecular strategies.

Today, more than ever, undeniable progress has been made in the field of artificial intelligence (AI). While it is impossible to predict all the applications and implications of these tools, AI has already improved protein identification rates in MS/MS analysis software. Tools such as Prosit and DIA-NN [44] are examples of AI-powered algorithms that have significantly enhanced spectrum prediction and peptide identification in data-independent acquisition workflows, often outperforming classical search engines. AlphaFold [45], another AI-driven tool, has revolutionized structural proteomics by providing high-accuracy protein structure predictions, enabling better functional annotation and interaction modeling. While traditional software like MaxQuant [46] and Proteome Discoverer remain foundational in proteomics workflows, these AI-integrated tools are emerging as complementary or even integrative components of next-generation analysis pipelines.

To take this a step further, AI must help unify and interpret the vast amounts of data generated, with the ultimate goal of identifying potential molecular targets and corresponding drugs to improve fungal control, anticipate resistance, and design sustainable, cost-effective control strategies [47]. This also requires addressing the persistent issue of inconsistent protein identifiers across databases by adopting standardized reference proteomes and robust cross-referencing tools to ensure comparability across studies. The challenge lies not only in interpreting proteomic data but also in integrating all available “-omic” data—genomics, transcriptomics, proteomics, and metabolomics—while establishing stronger links between them. A holistic approach will provide a more comprehensive understanding and enhance our capacity for modeling, ultimately leading to more effective strategies for combating *B. cinerea*.

AI is also expected to enhance our ability to differentiate the proteome of multiple species within the same sample, a field known as metaproteomics. This emerging discipline could provide a broader understanding of fungus–host interactions at a molecular level while also integrating their interactions with other organisms. Such advancements have the potential to pave the way for new drug discoveries or biological control strategies. In this sense, the first metaproteomic studies of *B. cinerea* infecting grapes have been initiated recently, showing the potential use of metaproteomics to deepen microbiota analysis and its role in plant–pathogen interaction [48].

In recent years, MALDI-TOF-MS technology has been successfully developed for the rapid identification of microorganisms, with the capability to identify bacteria down to the strain level. This technology relies on comparing the obtained microorganism spectra with a raw peak database of previously identified organisms. While this method is already widely used in the medical field, it also holds great potential for agricultural applications, particularly for detecting phytopathogens and implementing targeted control strategies. As mentioned earlier, this tool depends on spectral databases, meaning that the accuracy and speed of detection are directly linked to the completeness and reliability of these databases—an area that is still under development, especially for fungi. In *Botrytis*, first approaches have shown the capacity for a quick and easy determination of the *Botrytis* genus until the strain level [49].

## 5. Conclusions

*B. cinerea* is a highly aggressive phytopathogen that employs a wide array of strategies to infect and destroy its host. A deeper understanding of the molecular mechanisms underlying its pathogenicity is crucial for the development of novel strategies to control this fungus. As reviewed in this work, proteomics has undergone remarkable technological advances, enabling the detection of several thousands of proteins in a single experiment. Undoubtedly, this progress paves the way for the identification of new molecular targets for disease management.

However, in the context of *B. cinerea* and fungal phytopathology at large, the application of modern proteomic techniques remains in its early stages. The vast—and often overwhelming—volume of data generated highlights the urgent need to integrate robust bioinformatic approaches capable of extracting meaningful biological insights.

Several fundamental questions still need to be addressed to move the field forward. For instance, how can artificial intelligence aid in unifying protein databases for a single organism and improve the annotation of poorly characterized proteomes? Given that many identified proteins are associated with transitional stages, what is the proteomic landscape during stationary phases of fungal development? What methodologies could unlock the discovery of still undetected proteins, including those embedded in complex structures such as membranes, the complete transmembranome of which remains largely unexplored? Furthermore, which environmental factors are necessary to induce the expression of currently silent genes, and how can we enhance the detection of low-abundance proteins such as transcription factors in *B. cinerea*? What are the essential, specific, conserved, and host-independent pathways in *Botrytis cinerea*, which proteins compose these pathways, and which of them could serve as ideal molecular targets?

Beyond detection, a major challenge lies in deciphering the molecular strategies that allow *Botrytis* to reprogram its metabolism to adapt to different hosts. What molecular weaknesses in the plant does *B. cinerea* exploit to ensure successful colonization?

Addressing these questions will not only deepen our understanding of fungal pathogenicity but also guide the development of targeted and sustainable approaches to protect crops against this destructive organism.

## Figures and Tables

**Table 1:** Scientific publications involving *Botrytis cinerea* and proteomics released since last review in 2016.

Title	Author	Year	Ref.
Extracellular Vesicles of the Plant Pathogen *Botrytis Cinerea*	Dei Vallée, A. et al.	2023	[15]
The Adaptation of *Botrytis Cinerea* Extracellular Vesicles Proteome to Surrounding Conditions: Revealing New Tools for Its Infection Process	Escobar-Niño, A. et al.	2023	[16]
Identification of Virulence-Related Proteins during *Botrytis Cinerea*—Fruit Interaction at Early Phase	Liu, K. et al.	2023	[17]
The pH Regulator PacC: A Host-Dependent Virulence Factor in *Botrytis Cinerea*	Rascle, C. et al.	2018	[18]
Proteomic Analysis of Kiwifruit in Response to the Postharvest Pathogen, *Botrytis Cinerea*.	Liu, J. et al.	2018	[19]
PAMP Activity of Cerato-Platanin during Plant Interaction: An -Omic Approach	Luti, S. et al.	2016	[20]
Investigations on VELVET regulatory mutants confirm the role of host tissue acidification and secretion of proteins in the pathogenesis of *Botrytis cinerea*	Müller, N. et al.	2018	[21]
Unravelling the Initial Triggers of *Botrytis Cinerea* Infection: First Description of Its Surfactome.	Escobar-Niño, A. et al.	2019	[22]
iTRAQ-based proteomic analysis reveals the mechanisms of *Botrytis cinerea* controlled with Wuyiencin	Shi, L. et al.	2019	[23]
Impact of an Antifungal Insect Defensin on the Proteome of the Phytopathogenic Fungus *Botrytis cinerea*	Aumer, T. et al.	2020	[24]
Actin is Required for Cellular development and Virulence of *Botrytis cinerea* via the mediation of Secretory Proteins	Li, H. et al.	2020	[25]
Proteomic Study of the Membrane Components of Signalling Cascades of *Botrytis Cinerea* Controlled by Phosphorylation	Escobar-Niño, A. et al.	2021	[26]
Deciphering the Dynamics of Signaling Cascades and Virulence Factors of *B. Cinerea* during Tomato Cell Wall Degradation	Escobar-Niño, A. et al.	2021	[27]
